# Alternative Splicing Events and ABA Hormone Regulation in Drought Response of *Hippophae gyantsensis* L.

**DOI:** 10.3390/genes16030350

**Published:** 2025-03-18

**Authors:** Fanfan Lin, Yifan Cai, Shihai Yang, Yunqiang Yang

**Affiliations:** 1 Germplasm Bank of Wild Species, Yunnan Key Laboratory for Crop Wild Relatives Omics, Kunming Institute of Botany, Chinese Academy of Sciences, Kunming 650201, China; 2 CAS Key Laboratory of Tropical Plant Resources and Sustainable Use, Xishuangbanna Tropical Botanical Garden, Chinese Academy of Sciences, Menglun, Mengla, Xishuangbanna 666303, China; 3 University of Chinese Academy of Sciences, Beijing 100049, China; 4 Xizang Ecological Harmony Seed Industry Co., Ltd., Shigatse 857000, China

**Keywords:** *Hippophae gyantsensis*, alternative splicing, transcriptome, drought, ABA

## Abstract

(1) **Background**: *Hippophae gyantsensis*, a drought-tolerant plant native to the Tibetan Plateau, plays a crucial ecological and economic role. While its drought tolerance mechanisms have been extensively studied, the role of alternative splicing (AS) in drought resistance remains insufficiently explored. This study aims to elucidate how AS events regulate gene expression to enhance drought tolerance in *H. gyantsensis* under water-deficit conditions. (2) **Methods**: *H. gyantsensis* plants were subjected to progressive drought stress followed by rehydration. Physiological responses, transcriptomic data, and hormonal profiles were analyzed to investigate the plant’s adaptive mechanisms to drought stress, with a particular focus on abscisic acid (ABA) signaling-related genes. (3) **Results**: The results showed that *H. gyantsensis* maintained high leaf water content even under severe drought stress, emphasizing its strong drought resistance. A transcriptomic analysis revealed 11,962 differentially expressed genes, primarily enriched in hormone signaling and metabolic pathways. Notably, the accumulation of ABA was closely associated with AS events in ABA-related genes, such as *ZEPs*, *ABCG*, and *PP2C*. These genes produced multiple splice variants, indicating their role in modulating the ABA signaling pathway and enhancing drought tolerance. (4) **Conclusions**: This study highlights the pivotal role of AS in ABA signaling and drought tolerance in *H. gyantsensis*. It provides new insights into how AS contributes to plant adaptation to drought stress, bridging the knowledge gap in drought resistance mechanisms and emphasizing the importance of AS in plant stress responses.

## 1. Introduction

Sea buckthorn (*Hippophae* spp.) is a plant of considerable ecological and economic value, widely distributed across the temperate regions of Eurasia, particularly in resource-rich areas such as the Tibetan Plateau and Loess Plateau in China [1]. *Hippophae gyantsensis*, a key species within the *Hippophae* genus, is predominantly found on the Tibetan Plateau and exhibits exceptional tolerance to drought, cold, and saline–alkali conditions [2]. These unique traits not only enable its survival in extreme environments but underscore its potential for ecological restoration and revegetation in arid regions [3]. The arid environment of the Tibetan Plateau presents severe challenges for plant growth and survival. However, *H. gyantsensis* demonstrates strong drought tolerance through a range of physiological and morphological adaptations. Morphologically, its leaves possess well-developed palisade tissue, which effectively reduces water loss through transpiration and enhances adaptation to drought conditions [4,5]. In addition, sea buckthorn has a well-developed root system that extends deep into the soil, allowing it to access water from lower layers and enhancing its water acquisition capacity under drought conditions. *H. gyantsensis* adapts to drought stress through mechanisms such as regulating cell membrane permeability and accumulating osmotic adjustment substances. Under drought conditions, the proline and soluble sugar content in *H. gyantsensis* leaves increase significantly [6]. The accumulation of these substances lowers cellular water potential, improving the plant’s ability to absorb water and mitigating the impact of drought on cellular water balance. From an ecological perspective, the exceptional drought tolerance of *H. gyantsensis* makes it an ideal plant resource for vegetation restoration in arid and semi-arid regions [7]. Its extensive root system and efficient nitrogen-fixing capability not only contribute to soil improvement but play a crucial role in combating desertification and preventing soil erosion in severely affected areas.

*H. gyantsensis* also relies on abscisic acid (ABA) signaling pathways as part of its drought resistance mechanisms [8]. Studies have shown that ABA, a plant hormone, enhances drought tolerance by regulating stomatal closure, promoting the accumulation of osmotic adjustment substances, and activating the antioxidant defense system [9]. Under drought stress, ABA levels increase and bind to pyrabactin resistance protein/pyrabactin like protein/regulatory components of the ABA receptor (PYR/PYL/RCAR), activating sucrose non-repressible kinase 2 (SnRK2). This activation leads to the phosphorylation of transcription factors, such as the ABA-binding factor and the ABA-responsive element binding protein, as well as functional proteins, thereby inducing the expression of drought-responsive genes [10]. In *H. gyantsensis*, activation of the ABA signaling pathway regulates stomatal movement and reduces water loss through transpiration, helping to maintain water balance within the plant [11,12]. The ABA signaling pathway enhances drought tolerance by inducing the synthesis of osmolytes, such as proline and soluble sugars, which regulate the accumulation of osmotic adjustment substances, stabilize cellular structures, lower cell water potential, and ultimately improve the plant’s drought resistance [13]. Gene expression analyses indicate that, under drought stress, the expression of ABA signaling pathway-related genes, including ABA receptor genes, SnRK2 kinase genes, and downstream transcription factor genes, undergoes significant changes [14]. These gene expression changes are closely associated with drought tolerance, highlighting the critical regulatory role of the ABA signaling pathway in the drought resistance mechanisms of *H. gyantsensis*.

Alternative splicing (AS) events in genes related to the ABA signaling pathway significantly enhance plant drought tolerance through various mechanisms under drought stress [15]. AS is a process in which precursor mRNA from a single gene is spliced in different ways to produce multiple mature mRNAs, increasing the diversity and complexity of gene expression and regulating plant responses to drought stress [16]. Within the ABA signaling pathway, AS events can modulate the functions of ABA receptors and signal transduction proteins. Research has shown that, in *Arabidopsis*, the splicing factor SR45 is dephosphorylated under ABA stress, leading to its accumulation and modulation of the plant’s sensitivity to ABA [17]. The interplay between the ABA signaling pathway and AS plays a crucial role in regulating stomatal movement during plant responses to drought stress. Different splice variants generated through AS can regulate the expression of stomatal closure-related genes (e.g., *SLAC1*), reducing transpiration and helping to maintain internal water balance in plants [18]. Moreover, AS may influence the activity of ABA biosynthetic enzymes, thereby regulating ABA synthesis levels and enhancing drought perception and response in plants. In wheat, studies have shown that *TaPYL9* genes in the ABA signaling pathway enhance drought tolerance by regulating downstream TabZIP1 transcription factors, which activate genes involved in osmolyte synthesis and stomatal movement regulation [19]. These findings suggest that AS events within the ABA signaling pathway significantly improve plant survival under drought stress by increasing the diversity and functionality of gene expression [20]. In addition to the central role of the ABA signaling pathway in plant drought resistance, studies have demonstrated that strigolactones (SLs) contribute to drought responses by coordinately regulating stomatal behavior, root architecture, and antioxidant defense systems [21,22]. SLs not only enhance ABA biosynthesis and signal transduction [23,24] but improve plant drought adaptability by modulating these physiological processes [25,26]. These findings indicate that SLs play a role in ABA-mediated drought regulation, offering new insights into plant drought resilience mechanisms.

Although sea buckthorn is widely recognized as a drought-tolerant plant with well-studied adaptability to arid environments, the specific mechanisms underlying its drought resistance, particularly the contribution of AS events, remain incompletely understood. Current research primarily focuses on the mechanisms by which sea buckthorn adapts to drought, such as regulating membrane permeability, accumulating osmotic adjustment substances, and activating antioxidant enzyme systems [27,28]. However, the role of AS events in ABA signaling pathway genes in sea buckthorn’s drought resistance remains relatively unexplored. In this study, we subjected *H. gyantsensis* to drought treatments and integrated second- and third-generation transcriptome sequencing data to systematically investigate the influence of AS events on the drought adaptation of *H. gyantsensis* under varying drought conditions. Our findings provide substantial data support and a critical molecular foundation for a deeper understanding of sea buckthorn’s drought resistance mechanisms. In addition, they offer valuable insights for identifying potential targets for the genetic improvement of drought-tolerant crops.

## 2. Materials and Method

### 2.1. Plant Materials and Treatments

*H. gyantsensis* plants were grown in sanitized pots filled with a substrate consisting of vermiculite and loam soil in a 1:10 ratio by volume. The plants were cultivated under greenhouse conditions (25 °C/20 °C day/night temperature, relative humidity: 60–70%). During the first three months, all plants were irrigated to maintain full soil water holding capacity. Subsequently, they were subjected to a progressive water deficit treatment without manual watering adjustments. Samples were collected on days 30 and 40 of drought treatment and after 7 days of rehydration. Control plants were fully irrigated, with samples collected at the same time points. Each experimental group included three biological replicates, as described by Suseela et al. [29]. Collected leaf samples were wrapped in aluminum foil, immediately flash-frozen in liquid nitrogen, and stored at −80 °C for downstream molecular and biochemical analyses.

### 2.2. Determination of Drought-Related Physiological Indices

The leaf and soil relative water content (RWC) of *H. gyantsensis* seedlings were determined using the following formula: RWC (%) = [(fresh weight (Fw)) − (dry weight (Dw))]/[(turgid weight (Tw)) − Dw] × 100. To measure Fw, leaves were weighed immediately after collection. Tw represents the turgid weight of the tissue after soaking in water for 12 h at room temperature, while Dw is the dry weight [30]. Malondialdehyde (MDA) content was quantified using the MDA content assay kit (R21874-100T; Shanghai Yuan Ye Biotechnology Co., Ltd.; Shanghai, China). To prepare the MDA extract, 0.4–1 g of plant tissue was weighed and homogenized in tissue homogenizing solution at a 1:10 (g: mL) ratio. The homogenate was then centrifuged at 4000× *g* for 10 min, and the supernatant was collected as the MDA extract. The thiobarbituric acid (TBA) working solution was prepared according to the kit instructions. In the blank control, 200 μL of tissue homogenate, 1 μL of antioxidant, and 200 μL of TBA working solution were mixed. In the sample group, 200 μL of MDA extract, 1 μL of antioxidant, and 200 μL of TBA working solution were mixed. and placed in a 95 °C water bath for 30 min, ensuring no liquid spillage. After heating, the samples were allowed to cool to room temperature. They were then centrifuged at 4000× *g* for 10 min, and the supernatant was collected. Afterward, the absorbance of the supernatant was measured at 450 nm, 532 nm, and 600 nm using a spectrophotometer. The data were recorded, and the MDA concentration and content were calculated using the following formulas: MDA concentration (μmol/L) = 6.45 × (A_532_ − A_600_) − 0.56 × A_450_, MDA content (μmol/mg) = MDA concentration (μmol/L) × MDA extract volume (mL)/Fresh weight of plant tissue (g), where A_532_ = absorbance at 532 nm, A_600_ = absorbance at 600 nm, A_450_ = absorbance at 450 nm. Each experimental sample should be analyzed with three biological replicates.

### 2.3. Next-Generation Transcriptome Sequencing and Analysis

Total RNA was extracted from the stems and leaves of *H. gyantsensis* under different drought treatments using the CTAB-LiCl method [31]. The specific procedure is as follows: First, leaf tissue was ground and mixed with pre-warmed extraction buffer, followed by incubation at 65 °C. Next, a chloroform–isoamyl alcohol mixture was added, and the mixture was centrifuged to separate the layers. The supernatant was collected and LiCl was added to precipitate the RNA. The RNA pellet was washed with ethanol and dried, followed by DNase treatment to remove DNA. Finally, the RNA was re-suspended in RNase-free water. RNA concentration and purity were measured using a NanoDrop 1000 spectrophotometer (Nextomics Biosciences Co., Ltd.; Wuhan, China). High-quality RNA was enriched for mRNA using the Dynabeads™ mRNA Purification Kit (Thermo Fisher Scientific; Waltham, MA, USA), followed by random fragmentation and reverse transcription into cDNA. The cDNA library was constructed through end repair, A-tailing, and adapter ligation. After quality control, the library was sequenced on the DNBSEQ-T7 platform with paired-end 150 bp reads to generate raw transcriptome data. The data were quality-filtered using fastp software (version 0.23.2) to obtain clean reads. Gene expression levels were quantified using RNA-Seq by Expectation-Maximization (RSEM) [32] based on the reference genome index. Differentially expressed genes (DEGs) were identified using the DESeq2 package in R (v1.44.0) [33], with the criteria of *p*-value < 0.05 and |log2(fold change)| ≥ 1. Finally, gene ontology (GO) enrichment analysis was performed for the DEGs, and results were visualized using TBtools (version v2.154) [34].

### 2.4. Hormone Quantification

The contents of plant hormones, including ABA, zeatin (ZT), salicylic acid (SA), and jasmonic acid (JA), were determined using ultra-high-performance liquid chromatography instrument (Waters Corporation; Milford, MA, USA) coupled with a quadrupole-linear ion trap tandem mass spectrometry system (UHPLC-QTRAP-MS/MS) [35,36]. Chromatographic conditions were as follows: An ExionLC™ AD system equipped with a Waters BEH C18 column (2.1 mm × 100 mm, 1.7 μm) was used, with a column temperature of 40 °C, an injection volume of 2 μL, and a mobile phase consisting of 0.1% formic acid in water (A) and 0.1% formic acid in acetonitrile (B). The flow rate was 0.3 mL/min with gradient elution. Mass spectrometry conditions were set on an AB Sciex QTRAP 6500+ system operated in polarity-switching mode, with curtain gas (CUR) at 35 psi, collision gas (CAD) set to medium, ion spray voltage (IS) at 5500/−4500 V, source temperature (TEM) at 450 °C, and nebulizer gas (GS1) and heater gas (GS2) both at 40 psi. Plant hormone standards were prepared in 50% acetonitrile–water solution with a concentration range of 0.1–2000 ng/mL. A linear regression standard curve was constructed by plotting the mass spectrometric peak area (y-axis) against the standard concentration (x-axis). Sample preparation involved weighing 500 mg of sample, adding 400 μL of extraction solution (methanol–water = 4:1), followed by cryogenic grinding (−10 °C, 50 Hz, 6 min), low-temperature ultrasonication (5 °C, 40 KHz, 30 min), and incubation at −20 °C for 30 min. The samples were then centrifuged at 13,000 rpm for 30 min at 4 °C, and the supernatant was collected for analysis. The concentration of plant hormones was calculated using a linear equation, and the hormone content (μg/g) was determined using the formula: HC (μg/g) = C × V/W, where HC denotes the hormone content, C denotes the measured concentration, V is the sample volume before injection (400 μL), and W denotes the sample weight (0.5 g). Each experimental setup included three technical replicates.

### 2.5. Full-Length Transcriptome Sequencing and Analysis

Total RNA was extracted from *H. gyantsensis* samples and used to construct cDNA libraries according to the standard protocol of Oxford Nanopore Technologies (ONT), with 1 μg of total RNA per sample. The cDNA libraries were loaded onto a FLO-MIN109 flow cell and sequenced on the PromethION 24 platform (Oxford Nanopore Technologies, Oxford, UK) to generate long-read sequencing data. Raw reads were aligned to the reference *H. gyantsensis* genome [4] using the FLAIR toolkit (version v2.0.0). Full-length transcripts were identified with the align module while splicing sites were optimized using the correct module by integrating genome annotations and short-read sequencing data to enhance accuracy [37]. High-confidence transcript reference sequences were obtained through clustering and merging via the collapse module. Isoform expression levels were quantified using the quantify module, and differential expression along with AS events were analyzed with the diffExp and diffSplice modules, respectively. The results were visualized for interpretation and further downstream analysis.

## 3. Results

### 3.1. Response of H. gyantsensis to Drought Stress

**Figure 1:**
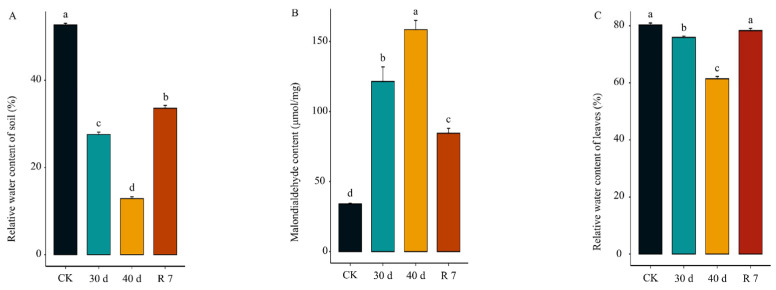
Physiological indices of *H. gyantsensis* under drought stress. (**A**) Comparison of relative soil water content. (**B**) Effect of drought stress on the leaf Malondialdehyde (MDA) content in *H. gyantsensis*. (**C**) Relative water content of leaves under control and drought stress conditions. Data were analyzed via one-way analysis of variance (ANOVA) followed by Tukey’s test. Error bars indicate SE. Means with different letters are significantly different (*p* < 0.05). Treatment groups: CK (control: optimal irrigation); 30 d (30−day progressive drought); 40 d (40−day sustained drought); R 7 (7−day post-stress rehydration).

To assess the drought tolerance of *H. gyantsensis*, we subjected the plants to varying drought treatments. As drought progressed, the soil water content declined to 27.58% after 30 days of water deficit (Figure 1A), while the relative electrolyte leakage in drought-treated plants increased significantly compared with the leakage in control plants (Figure 1B). Although leaf water content in drought-treated plants decreased relative to the control group, it remained at a relatively high level of 75.92% under drought conditions (Figure 1C). These findings indicated that *H. gyantsensis* leaves exhibited a physiological response to drought stress and demonstrated strong drought tolerance. After 40 days of drought treatment, soil water content further declined to 12.87%, yet *H. gyantsensis* maintained a leaf water content of 61.43%, reinforcing its drought resistance. In addition, a rehydration experiment conducted after 40 days of drought revealed a significant decrease in relative electrolyte leakage after 7 days of rehydration, accompanied by an increase in the leaf water content compared with drought conditions. These results highlight the strong drought resistance of *H. gyantsensis*.

### 3.2. Transcriptional Changes in H. gyantsensis Under Drought Stress

**Figure 2:**
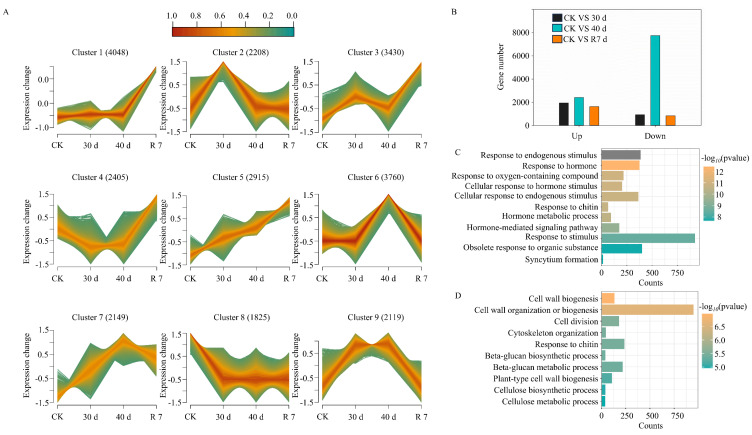
Transcriptomic analysis of differentially expressed genes (DEGs) in *H. gyantsensis* under drought stress. (**A**) Clusters of expressed genes in leaves under drought treatment. (**B**) Number of DEGs significantly up- or downregulated at the transcriptional level among 30 day/CK, 40 day/CK, and R 7/CK comparisons. (**C**) GO enrichment analysis of the DEGs in the 30-day drought treatment. (**D**) GO enrichment analysis of the DEGs in the rehydration treatment. Treatment groups: CK (control: optimal irrigation); 30 d (30−day progressive drought); 40 d (40−day sustained drought); R 7 (7 −day post-stress rehydration).

To further investigate the molecular basis of *H. gyantsensis* responses to drought stress, we conducted a next-generation RNA-Seq transcriptome analysis on *H. gyantsensis* leaves and stems under normal conditions, as well as after drought and rehydration treatments (Table S1). Through an analysis of the temporal expression patterns of genes throughout the drought and rehydration processes, nine distinct expression patterns were identified in the leaves. In pattern 2, 2208 genes exhibited high expression at 30 days of drought but subsequently decreased. In pattern 6, 3760 genes were significantly activated after 40 days of drought treatment. In patterns 7 and 9, drought induced sustained high-level expression of 2149 and 2119 genes, respectively, which then decreased after rehydration (Figure 2A). By referencing the *H. gyantsensis* genome sequence [4], we compared the expression patterns of the DEGs before and after drought and rehydration using DESeq (*p*-value < 0.05) with |log_2_ (fold-change)| ≥ 1. Compared with the control plants, 2866 DEGs were identified in plants subjected to 30 days of drought, with the number of upregulated genes exceeding that of downregulated genes (Figure 2B). After 40 days of drought treatment, the number of DEGs increased to 10,159, including 2407 upregulated and 7752 downregulated genes. Following 7 days of rehydration, 2461 DEGs were detected. To further analyze the functional significance of these DEGs, we performed a GO enrichment analysis (*p* < 0.05) under drought and rehydration conditions. The results revealed that, at 30 days of drought, biological processes related to the response to endogenous stimuli, response to hormones, response to oxygen-containing compounds, cellular response to hormone stimuli, and hormone-mediated signaling pathways were significantly enriched (Figure 2C). On day 40 of drought stress, the enriched terms included response to oxygen-containing compounds, response to chitin, β-glucan metabolic process, and cellulose metabolic process (Figure 2D). These results suggest that plant hormones play an active role in the response to drought stress in *H. gyantsensis* at 30 days of drought, while metabolite biogenesis becomes more prominent in the plant adaptation after 40 days of drought.

### 3.3. Plant Hormone Response in H. gyantsensis Under Drought Stress

**Figure 3:**
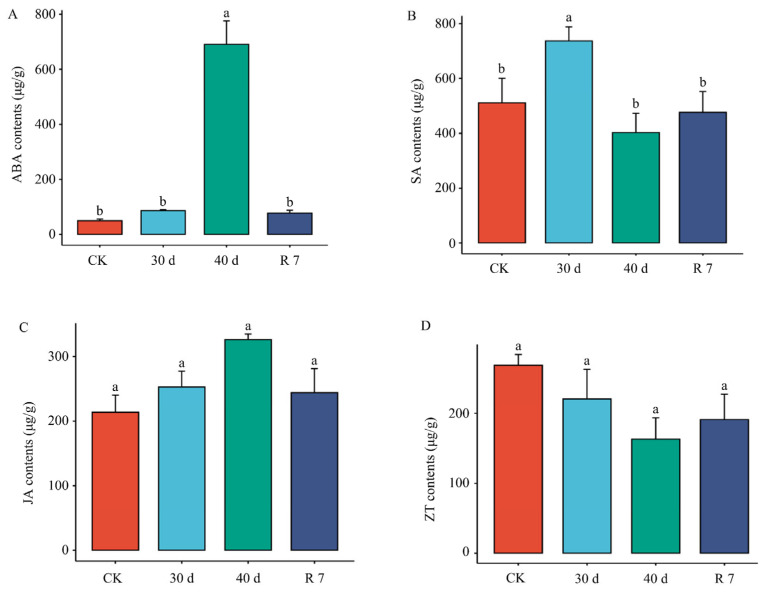
Effects of drought stress on hormone levels in *H. gyantsensis* seedlings. The panels (**A**–**D**) present the measured levels of the hormones ABA, SA, JA, and ZT, with the x-axis representing different treatment groups and the y-axis indicating the corresponding hormone concentrations. Data represent mean values from three independent measurements and were analyzed using one-way analysis of variance (ANOVA) followed by Tukey’s test. Different letters indicate significant differences (*p* < 0.05). Abbreviations: ABA, abscisic acid; SA, salicylic acid; JA, jasmonic acid; ZT, zeatin. Treatment groups: CK (control: optimal irrigation); 30 d (30−day progressive drought); 40 d (40−day sustained drought); R 7 (7−day post-stress rehydration).

According to the significant enrichment of genes related to plant hormone signaling pathways under drought conditions, we further analyzed the levels of plant hormones ABA, SA, JA, and ZT during drought and rehydration treatments (Figure 3A–D). The results revealed varying degrees of hormonal changes in response to drought. Among these, ABA, a key hormone involved in drought resistance, showed a significant increase after 40 days of drought, reaching more than 7.9 times the control level. However, after 7 days of rehydration, the ABA levels sharply declined to near-control levels. These findings suggest that the ABA signaling pathway, along with other plant hormones, plays a crucial role in regulating the response of *H. gyantsensis* to drought stress.

### 3.4. Alternative Splicing Events in Response to Drought Stress

**Figure 4:**
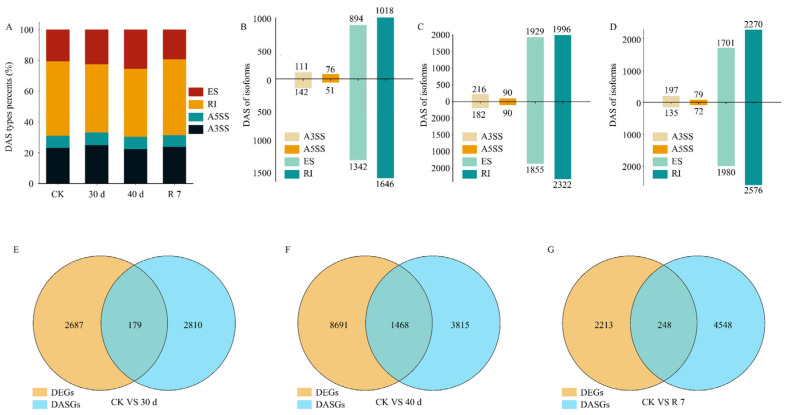
Analysis of differentially alternative splicing (DAS) events based on full-length transcriptome data. (**A**) Predicted alternative splicing events in different groups under control and drought stress conditions. (**B**–**D**) Number of isoforms from differentially alternative splicing (DAS) isoforms that were up- or downregulated at the transcriptional level among 30 day/CK, 40 day/CK, and R 7/CK comparisons. (**E**–**G**) Comparison of differentially expressed genes (DEGs) and differentially alternative splicing genes (DASGs) among 30 day/CK, 40 day/CK, and R 7/CK. AS types: A3SS, Alternative 3′ Splice Site; A5SS, Alternative 5′ Splice Site; ES, Exon Skipping; RI, Retained Intron. Treatment groups: CK (control: optimal irrigation); 30 d (30−day progressive drought); 40 d (40−day sustained drought); R 7 (7−day post-stress rehydration).

Studies have shown that AS events in plant hormone signaling pathway genes play a crucial role in regulating plant drought tolerance [38]. To investigate AS events in *H. gyantsensis* under drought stress, we conducted a full-length transcriptome analysis (Table S2) and compared differential alternative splicing (DAS) events across drought treatments. Our results revealed significant changes in the proportion of DAS types at 30 and 40 days of drought compared with the control. A total of 5280, 8680, and 9010 DAS isoforms were identified under drought and rehydration conditions., respectively, with Retained Intron (RI) events being the main AS type under drought treatments. (Figure 4A–D). Further analysis of the integrating DEGs and differentially alternative splicing genes (DASGs) results revealed that 179, 1468, and 248 genes exhibited both DAS events and differential expression under drought and rehydration conditions (Figure 4E–G). These findings suggest that drought stress induced the production of additional AS isoforms in *H. gyantsensis*. The genes undergoing DAS events responded to drought stress by modifying transcript levels and transcript variants.

### 3.5. Drought Response of Alternative Splicing Events in the ABA Signaling Pathway

**Figure 5:**
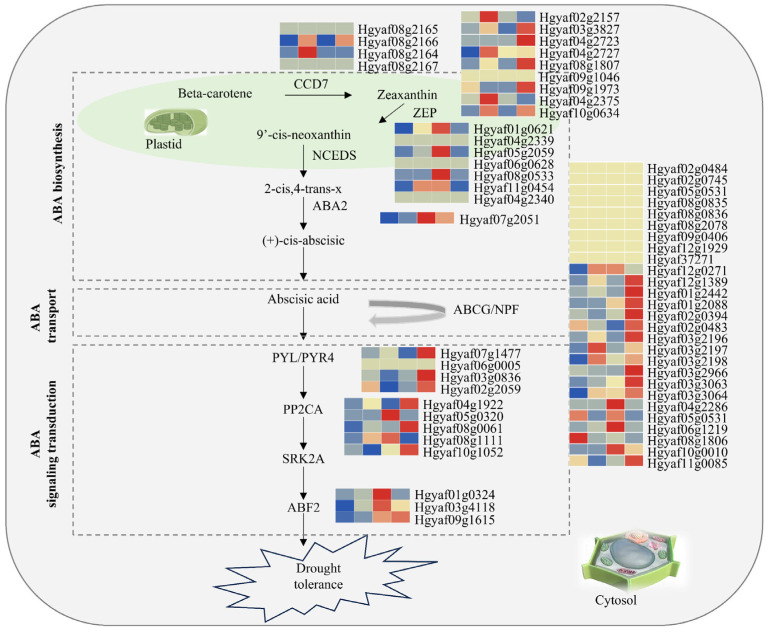
Analysis of genes involved in the ABA biosynthesis pathway in *H. gyantsensis*. The ABA metabolic pathway and expression patterns of genes related to the ABA metabolism under control and drought stress conditions [7,8] Key enzymes in ABA biosynthesis: CCD7 (carotenoid cleavage dioxygenase 7); ZEP (zeaxanthin epoxidase); NCEDS (9-cis-epoxycarotenoid dioxygenase). ABA transport regulation: ABA: ABCG/NPF (ATP-binding cassette G/nitrate–peptide transporter family). Essential elements in the ABA signaling pathway: PYL/PYR4 (PYR1-like/pyrabactin resistance 4); PP2CA (protein phosphatase 2CA); SRK2A (sucrose non-fermenting 1-related protein kinase 2A); ABF2 (ABA-responsive element-binding factor 2).

**Figure 6:**
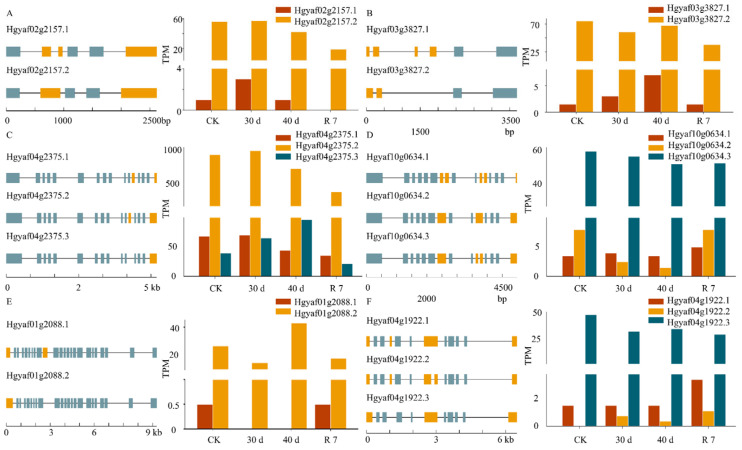
Schematic representation of genes involved in alternative splicing (AS) within the ABA metabolic pathway. (**A**–**F**) boxes represent exons, and lines represent introns; the expression levels of different splice variants were determined based on third-generation full-length transcriptome data. Treatment groups: CK (control: optimal irrigation); 30 d (30−day progressive drought); 40 d (40−day sustained drought); R 7 (7−day post-stress rehydration). Expression units: TPM (transcripts per million).

To further investigate the role of the ABA signaling pathway in the drought response of *H. gyantsensis*, we identified and analyzed the expression levels and AS events of key regulatory genes within the pathway. The results revealed distinct changes in the expression levels of genes involved in ABA biosynthesis, transport, and signal transduction under drought stress (Figure 5). The expression levels of *NCED* genes (Hgyaf01g0621 and Hgyaf05g2059), which play a crucial role in the ABA biosynthesis pathway, increased sharply after 30 days of drought treatment and remained elevated after 40 days, before decreasing following rehydration. In addition, *PP2CA* genes (Hgyaf05g0320 and Hgyaf08g1111), which are involved in ABA signal transduction, also exhibited an upregulation trend under drought stress compared with control conditions. Furthermore, we observed AS events in *ZEP* (Hgyaf02g2157, Hgyaf03g3827, Hgyaf04g2375, and Hgyaf10g0634)*, ABCG* (Hgyaf01g2088), and *PP2CA* (Hgyaf04g1922) genes under drought stress (Figure 6A–F). The *ZEP* genes generated additional transcript isoforms through AS, while *ABCG* (Hgyaf01g2088) exhibited two distinct AS variants, with splice variant 2 becoming the predominant form under drought conditions (Figure 6E). These findings suggest that the formation of specific isoforms through AS plays a role in the ABA signaling pathway drought response in *H. gyantsensis*, potentially influencing gene function under drought stress.

## 4. Discussion

The ecological functions of *H. gyantsensis* on the Tibetan Plateau are closely tied to its drought tolerance, making it crucial for soil and water conservation and vegetation restoration. The ability of *H. gyantsensis* to withstand drought not only ensures its survival in arid environments but contributes to ecosystem stability and biodiversity conservation by improving soil structure and increasing vegetation cover. In this study, our results demonstrated that the leaf water potential of *H. gyantsensis* remained relatively high under drought stress, a response similar to that observed in other *Hippophae* species [39]. These findings suggest that *Hippophae* species may enhance drought tolerance by regulating intracellular water balance to sustain physiological functions under drought conditions.

Studies have demonstrated that the upregulation of genes involved in the ABA biosynthetic pathway promotes ABA accumulation in plants, thereby enhancing drought tolerance. As a key gene in ABA biosynthesis, the overexpression of *AtNCED3* in *Arabidopsis* significantly increases ABA levels, thereby enhancing plant drought resistance [40]. In cotton, *GhCYP94C1* regulates the expression of *GhNCED9* in virus-induced gene silencing (VIGS) mutants, leading to its significant upregulation under drought stress and promoting ABA accumulation, which aligns with the observed strong upregulation of *VuNCED1* in cowpea leaves under drought conditions [41,42]. Additionally, studies have suggested that the SL signaling pathway also modulates the expression of *NCED* genes, thereby influencing ABA synthesis and accumulation [43]. Consistently, SL biosynthesis or signaling mutants (e.g., max1 and max2) exhibit significant downregulation of *NCED3* gene expression, resulting in reduced ABA levels and compromised drought tolerance [44,45]. Similarly, our findings further confirm that the upregulation of *NCED* gene expression indeed promotes ABA biosynthesis and accumulation in plants, significantly enhancing their drought resistance. However, the specific regulatory mechanisms of *NCED* genes within the context of our study remain to be further explored and elucidated. Furthermore, this study revealed substantial differences in the gene expression levels of *H. gyantsensis* under varying drought conditions. After 30 days of drought stress, the DEGs were predominantly enriched in plant hormone metabolic and signaling pathways, with a particular emphasis on ABA biosynthesis. In addition, the ABA accumulation in *H. gyantsensis* increased with the severity of drought stress, peaking after 40 days of drought treatment. These results suggest that drought stress induces changes in the expression levels of ABA signaling pathway genes, leading to increased ABA content, which may contribute to the drought resistance of *H. gyantsensis*.

AS events in the ABA signaling pathway play a crucial role in enhancing plant survival under drought stress by regulating gene expression diversity and function [46]. Under drought conditions, six ABA biosynthetic genes *ZEPs* (Hgyaf02g2157, Hgyaf03g3827, Hgyaf04g2375, and Hgyaf10g0634)*, ABCG* (Hgyaf01g2088)*,* and *PP2C* (Hgyaf04g1922), exhibited AS events in *H. gyantsensis*. The *PP2C* genes generated multiple splice variants, which may have distinct functional roles in plant responses to drought [16]. In maize, drought stress significantly suppresses the expression of *ZmPP2C26*, suggesting that it may function as a negative regulator of drought-responsive gene expression, thereby enhancing plant drought tolerance [47]. Interestingly, some studies have indicated that *PP2C* genes may also act as positive regulators under certain conditions [48]. The overexpression of *ZmPP2C2* in tobacco significantly enhances antioxidant enzyme activities (e.g., SOD, POD, and CAT), thereby improving drought tolerance. *ZmPP2C72* and *ZmPP2C97* are notably upregulated under drought, ABA, and NaCl treatments. Moreover, the overexpression of these genes in *Arabidopsis* enhances drought tolerance by activating antioxidant enzymes [49]. In *H. gyantsensis*, the *PP2C* (Hgyaf04g1922) gene produces three splice variants, with *PP2C.1* remaining relatively unchanged during drought stress, while *PP2C.2* shows increased expression after 30 days of drought treatment. This suggests that *PP2C.2* may function as a positive regulator in the drought response.

*ZEP* is a rate-limiting enzyme in the ABA biosynthesis pathway, catalyzing the epoxidation of zeaxanthin [50]. The expression level of the *ZEP* gene is upregulated under drought stress, resulting in increased ABA synthesis and enhanced drought tolerance in plants [51]. In addition, SLs indirectly influence the biosynthesis of ABA by regulating the expression of the *ZEP* gene. For example, in *Arabidopsis*, SL synthesis mutants (such as max2) exhibit downregulation of *ZEP* gene expression, resulting in a decrease in ABA levels, which in turn weakens the plant’s drought resistance [45]. Our study revealed for the first time that *ZEP* genes in *H. gyantsensis* exist in different splice variants, *ZEP.1*, *ZEP.2*, and *ZEP.3*. Among these, *ZEP.2* and *ZEP.3* were the predominant isoforms expressed in response to drought stress, suggesting their critical role in mediating drought adaptation mechanisms. However, the underlying mechanism requires further exploration and clarification. However, the specific regulatory mechanisms still require further investigation and clarification. Additionally, SLs may indirectly regulate ABA metabolism by influencing the AS or transcriptional expression of the *ZEP* gene, thereby participating in the drought tolerance regulation of *H. gyantsensis*. This mechanism still needs further research and validation.

## 5. Conclusions

In this study, we conducted transcriptome and AS events analyses using Illumina (extomics Biosciences Co., Ltd; Wuhan, China) and ONT (extomics Biosciences Co., Ltd; Wuhan, China) sequencing technologies to investigate the response of *H. gyantsensis* to drought stress. Increased expression levels were ob-served in the genes associated with plant hormone biosynthesis, particularly those involved in ABA biosynthesis. Notably, six genes in the ABA pathway, *ZEPs* (Hgyaf02g2157, Hgyaf03g3827, Hgyaf04g2375, and Hgyaf10g0634), *ABCG* (Hgyaf01g2088), and *PP2C* (Hgyaf04g1922), underwent AS events, which may contribute to the drought tol-erance of *H. gyantsensis*. The three splice variants of the *PP2C* gene, *PP2C.1*, *PP2C.2*, and *PP2C.3* exhibited distinct expression patterns under drought stress, with *PP2C.2* potentially acting as a positive regulator in the drought re-sponse. Furthermore, this study is the first to report the occurrence of AS in the *ZEP* gene under drought conditions. These findings suggest that AS events play a crucial role in the drought resistance mechanisms of *H. gyantsensis* by regulating gene expression diversity and function. This study provides new insights into the molecular mechanisms underlying plant responses to abiotic stress.

## Data Availability

The original contributions presented in the study are included in the article/Supplementary Materials, further inquiries can be directed to the corresponding author.

## Supplementary Materials

The following supporting information can be downloaded at: https://www.mdpi.com/article/10.3390/genes16030350/s1, Table S1: Statistics of Illumina sequencing data of H. gyantsensis. Table S2: Statistical summary of the full-length transcriptome sequences generated using the ONT platform.
